# Lessons learned about co-creation: developing a complex intervention in rural Peru

**DOI:** 10.1080/16549716.2020.1754016

**Published:** 2020-05-14

**Authors:** Maria Lazo-Porras, Silvana Perez-Leon, Maria Kathia Cardenas, M. Amalia Pesantes, J. Jaime Miranda, L. Suzanne Suggs, François Chappuis, Pablo Perel, David Beran

**Affiliations:** a Division of Tropical and Humanitarian Medicine, Geneva University Hospitals & University of Geneva, Switzerland; b CRONICAS Center of Excellence in Chronic Diseases, Universidad Peruana Cayetano Heredia, Lima, Peru; c School of Medicine, Universidad Peruana Cayetano Heredia, Lima, Peru; d Faculty of Communication Sciences, Università della Svizzera Italiana, Lugano, Switzerland; e Swiss School of Public Health, Zurich, Switzerland; f Department of Non-Communicable Disease Epidemiology, London School of Hygiene & Tropical Medicine, London, UK

**Keywords:** Co-creation, intervention development, diabetes, hypertension, neurocysticercosis

## Abstract

**Background**: Co-creation is the process of involving stakeholders in the development of interventions. Although co-creation is becoming more widespread, reports of the process and lessons learned are scarce.

**Objective**: To describe the process and lessons learned from using the COHESION manual, a co-creation methodology to develop interventions aimed at the improvement of diagnosis and/or management of chronic diseases at the primary healthcare level in a low-resource setting in Peru.

**Methods**: Observational study to describe the use of the COHESION manual ‘Moving from Research to Interventions: The COHESION Model’ developed for a multi-country project in low- and middle-income countries for co-creation and the adaptations needed to customize it to the local context of rural communities in northern Peru.

**Results**: The actual process of co-creation in Peru included co-creation-related questions in the formative research; an initial consultation with stakeholders at the micro, meso, and macro levels (e.g. community members, health workers, and policy-makers); the analysis of the collected data; a second consultation with each stakeholder group; the prioritization of intervention options; and finally the design of a theory of change for all activities included in the complex intervention. The complex intervention included: 1) offer training in specific diseases and soft skills to health workers, 2) create radio programs that promote chronic disease prevention and management plus empower patients to ask questions during their visits to primary health care (PHC) facilities, and 3) provide a small grant to the PHC for infrastructure improvement. Small adaptations to the COHESION manual were necessary for this co-creation process.

**Conclusion**: This study provides a practical example of the process of co-creating complex interventions to increase access and quality of health care in a low-resource setting. The process, components, challenges and opportunities identified could be useful for other researchers who want to co-create interventions with beneficiaries in similar settings.

## Background

Co-creation, also referred to as co-production, is described as ‘getting everybody around the table so you’re valuing the knowledge everybody has’ [1]. Co-creation is the process of involving stakeholders in the development of interventions, defining directions and purposes, and solving problems together [2,3]. It is one of many methods that promote the participation of stakeholders, community members, health workers, and policy-makers in public health interventions. Involving stakeholders in the planning and development of projects can facilitate the implementation process as well as the sustainability and scalability of interventions [4–6].

Globally, 85% of premature deaths due to non-communicable diseases (NCDs) occur in low- and middle-income countries (LMICs) [7,8]. In parallel people in these countries suffer from infectious diseases and vulnerable groups continue to be affected by Neglected Tropical Diseases (NTDs) [9]. Although health outcomes have improved in LMICs, high-quality health systems are still lacking. Poor quality of care is currently a larger barrier to reduce mortality than insufficient access to services [10]. In addition to dealing with the high prevalence of diseases, primary health care (PHC) suffers from limited human resources, the absence of diagnostic tools, and the low availability of medicines [11,12]. For these reasons, there is an urgent need for approaches that address complex problems related to access and quality of health care [10]. However, a recent systematic review of process evaluation studies of complex interventions focused on chronic diseases found a lack of alignment between local needs expressed by stakeholders and the interventions implemented [13]. It was also found that limited knowledge of the health system could affect the implementation of interventions. Thus, co-creation appears to be a promising and worthwhile approach in planning and designing interventions [14].

Although co-creation is becoming more widespread in health, reports of the process are scarce, and most of them come from the private sector and academia in high-income countries [15]. There are few examples of co-creation from the health sector, PHC and LMICs [16]. Thus, the objective of this paper is to present the lessons learned from the use and adaptation of co-creation methodology when developing a complex intervention aimed to improve the diagnosis and/or management of chronic disease in a rural area of Peru.

## Methods

This case study is based on the COmmunity HEalth System InnovatiON (COHESION) project, funded by the Swiss Programme for Research on Global Issues for Development [17]. The COHESION project hypothesised that sustainable, gender and context-appropriate interventions implemented at policy, health system, and community levels could be developed and integrated into PHC responses through a clear understanding of barriers and enablers for diagnosis, management, and care of NCDs and NTDs. The project was developed in Peru, Nepal, and Mozambique [17].

The project conducted formative research between 2016 and 2017, including a policy analysis, a Health System Assessment, and a community perception study (these studies will not be presented in this manuscript). Following this research, a co-creation methodology was developed and implemented during 2018 [18,19]. This paper describes the Peruvian experience in using and adapting that methodology where the disease focus was diabetes, hypertension, and neurocysticercosis.

### Setting

Peru is an upper-middle-income country located in South America. It has a population of 31 million with 20% living in rural areas [20]. This study was conducted in two rural communities in two districts of Piura, a region in northern Peru where 21% of its population lives in rural areas [21]. The districts, Ayabaca and Montero, include populations with limited access to public services and high rates of poverty (68% vs the national average of 21%) [21]. In each district, two PHCs, one medical center and one health post, were selected. Each PHC covers a population of 1069–1089 inhabitants.

### Participants

Participants in the co-creation process included: the project team; macro or national level actors (Ministry of health, policy-makers, key opinion leaders, organizations and central medical stores); meso or intermediate level actors (Ministry of Health office at the regional level, regional medical stores, pharmacies, hospitals and clinics); and micro or individual level actors (health workers, traditional healers, patients and caregivers, community representatives). Separate activities were conducted with the project team and each level of actors, as to focus discussions and decisions plus to maximize open participation.

### Co-creation method

The COHESION manual on co-creation [18] includes two consultations with stakeholders, data analyses, and a co-design phase. Data from the formative studies was analysed and the most relevant findings were synthetized and prepared for consultations with stakeholders. The first interactions included meetings with each actor separately to present and discuss the findings, exchange ideas, make proposals for possible interventions, and identify factors necessary to make the interventions successful and sustainable. The project team then assessed the proposed potential interventions. Interventions impacting only one of the levels (macro, meso or micro) were discarded as the goal of the project was to have an impact on different levels. Interventions that were priorities for all stakeholders, crossing macro, meso, and micro levels, that could be completed and evaluated with the scope and timeline of the project were kept for the second consultation.

During the second consultation (prioritization), the same stakeholders and project team discussed the list of interventions and assessed their level of priority. Following the manual, stakeholders completed a table with evaluation criteria listed [18,19]. From this list, the project team with assistance of the national project advisory board, applied the COHESION Score. The score took factors into account (impact, population benefitting, feasibility, sustainability and scalability) with the aim of providing a ‘score’ to each intervention for prioritising [19]. A selection of three interventions was made and subsequently discussed at a meeting with the multi-country project team.

Finally, during the co-design phase, the intervention was defined and designed using a participatory approach. Each team returned to their home countries with the three selected interventions.

The Theory of Change was discussed with stakeholders to test the intervention, refine the design, and gain buy-in. The final determination of the inputs needed, such as who is going to deliver the activities, how the intervention is going to be delivered and when, are details that will be decided with the stakeholders.

### Public involvement

The co-creation process is an approach that is only possible with the participation of stakeholders. As we described in previous sections; our participants from macro, meso and micro level were informants in the formative phase and then they were involved to define the components of the intervention and to co-design these components. In the future, during the implementation of the project the different stakeholders will be active participants in different steps.

## Results

**Figure 1.**
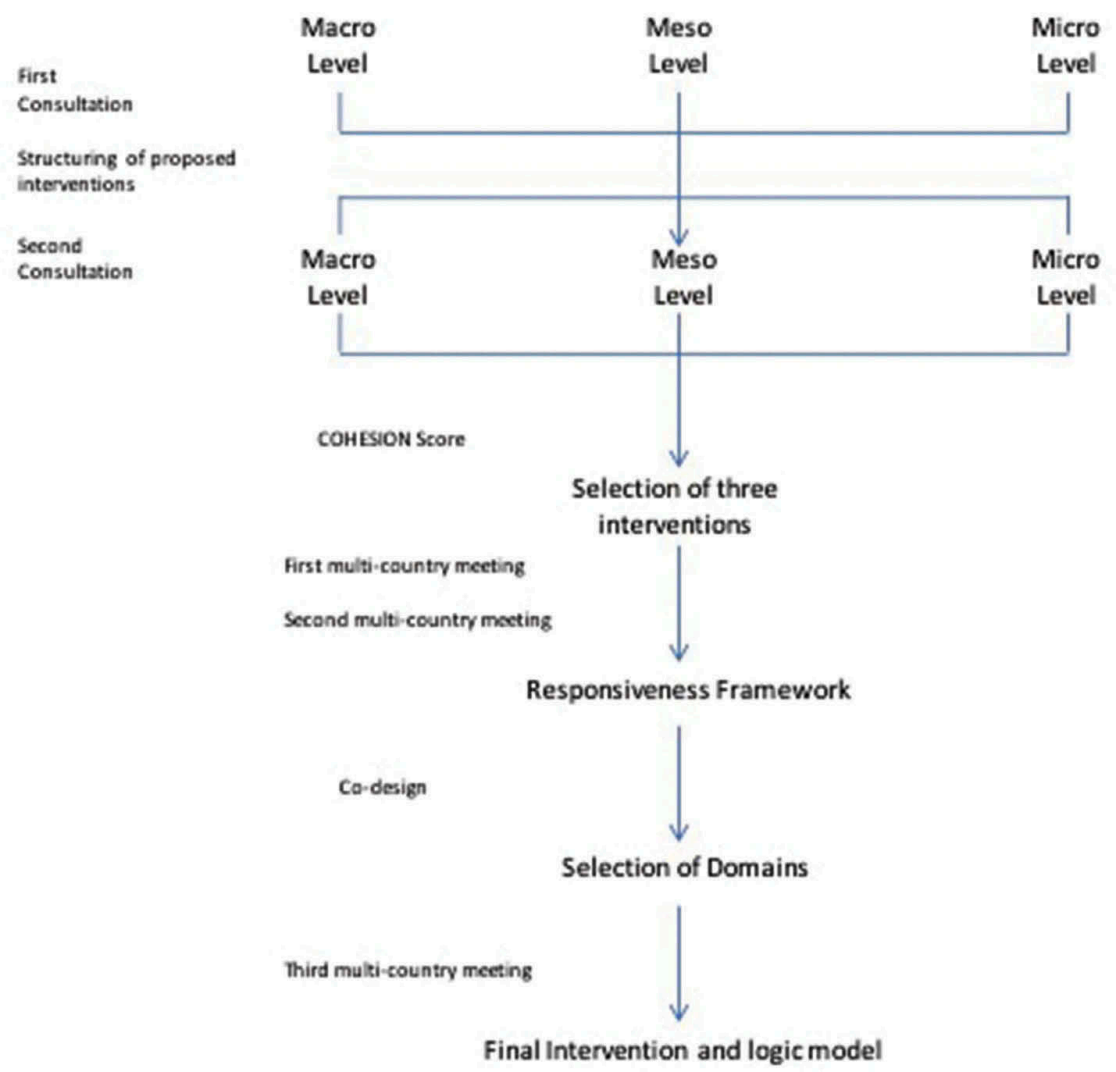
Overall Co-creation process in Peru

This project used the COHESION manual [18,19] to guide the co-creation process and made adaptations where necessary. These were included in co-creation activities but also when conducting the formative research prior to co-creation. Adaptations at each stage of the project are described below and presented in Table 1. The overarching process is presented in Figure 1.

**Table 1.** Adaptations to the manual of Co-creation

	What manual said	What was done in Peru	Adaptations and justification
First consultation	Review and analyse the formative studies (policy analysis, health system assessment and community perception study) and identify the most appropriate communication tools for sharing the results.	First consultation was held with some highlights of the formative studies but not with the complete analysis of the data.	The complete analysis of the data would take at least 6 months. That time would have prolonged the time of the co-creation process.
	Present the research findings to stakeholders with the aim of sharing insights and soliciting all possible interventions.	In the case of participants at the macro and meso level, one question was added to the face-to-face interviews that were part of the health system assessment.	Meet a group of stakeholders from the macro level is difficult because of their busy schedules. (A previous experience demonstrated that) In the case of the meso level, we met with them to present the project, but not to collect intervention proposals.
	Complete a table with the problems identified, provide a justification based on the results of the formative studies, and a list of potential interventions	In Peru, the table was not complete.	Instead of the table, we used a different approach during the meeting to promote participation. Also, some of the participants did not know how to write.
Analysis I: Structuring of proposed interventions	Assess the ‘pile’ of interventions and discard interventions listed as only impacting one of the areas	Proposals were reviewed and researchers applied some filters to joint similar proposals and selected some interventions for the second consultation	Project team realized that most of the proposals could have an impact in different levels. So discarded only proposals outside the scope of the project.
Second consultation	Rather than presenting to a large group of stakeholders from communities, the health system and policy level, choose people who are seen as leaders in their different stakeholder groups.	We invited all the previous participants from the first consultation and at community level, an open invitation was sent.	Include only leaders could have a low participations rate and probably a selected group of participants could be bias.
	Begin this process with the community completing a ranking tool that highlight the problem needed to be addressed, the intervention or interventions possible to address this problem and a ranking.	In small groups, participants were asked to select the three most important interventions and the three less important interventions and one intervention was left in the middle.	This approach was chosen because it was difficult to ranked seven interventions.
	Held separate meetings with stakeholders from the health system and policy level.	We sent an e-mail to the participants of the first consultation with information of the seven potential interventions and a table with evaluation criteria.	Like in the first consultation, meet a group or have an interview with stakeholders at the macro level is difficult because of their busy schedules.
Analysis II: Selection of 3 interventions	Select from the previous step the top 4 interventions from each group (community, health systems and policy level). This give a maximum of 12 interventions	Seven potential interventions were evaluated and three in each group (macro level, meso level and micro level – community and health workers-) were prioritised.	After the first consultation, researchers had only seven interventions, and those were prioritised.
	COHESION Team should score the different interventions proposed and the country’s Advisory Board can be involved in this process to provide another perspective.	Five members of the team gave a score to the interventions together and the Advisory Board did not participate in the process.	A meeting with the Advisory Board had been held previous to the second consultation, and they received their advice about the proposals. Their comments were considered to give the score during the evaluation.
First multi- country meeting	Arrive at the meeting with 3 selected interventions. Describe the three interventions using the following three criteria: Target, Action and Means.	In a meeting, the team worked on a preliminary version of TIDieR (what, who, how of the intervention)	It took longer than expected and so this work took place at the meeting rather than before.
Second multi-country meeting: Framework selection	Not include in the manual	This meeting was conducted with the coordinators and research assistants of each country (Peru, Nepal and Mozambique) and one Principal Investigator with support of two consultants with experience in development of interventions in chronic diseases.	
Co-design	Each team return to their home countries with the three selected interventions at the policy, health system and community levels. Discuss the Theory of Change with local stakeholders to test, help refine their design as well as gain buy-in.	The implementation of the co-design had a different method. During the co-design, we had separate meetings with each group of stakeholders (community, health workers and regional stakeholders) to present the framework (Responsiveness Framework) and the project team selected with them the most important dimensions of the framework that needed to be included in the intervention	At the macro level, we did not plan a meeting because the coordination with these participants had been always challenged and researchers considered that meso and micro level were more important for this part of the process.
Final interventions and development of a logic model	Not include in the manual	The project team worked on their logic models and then discussed in a co-design final meeting with stakeholders.	We found this important as a final step to understand what was expected of intervention activities (outputs, outcomes and impact) and what was required to develop them (inputs and activities).

### First consultation

One of the first differences between the proposed COHESION manual and their actual implementation in Peru was done ahead of the first consultation. The analysis of the results from the formative studies took longer than expected by the researchers. Therefore, the first consultation presented some highlights of the formative studies, instead of the complete analysis.

At the macro and meso level, one question was added to the face-to-face interviews that were part of the Health System Assessment of the formative research. The question was ‘In your opinion, what politics or projects could be implemented to improve the primary health care of chronic conditions/neurocysticercosis in rural areas?’

**Figure 2.**
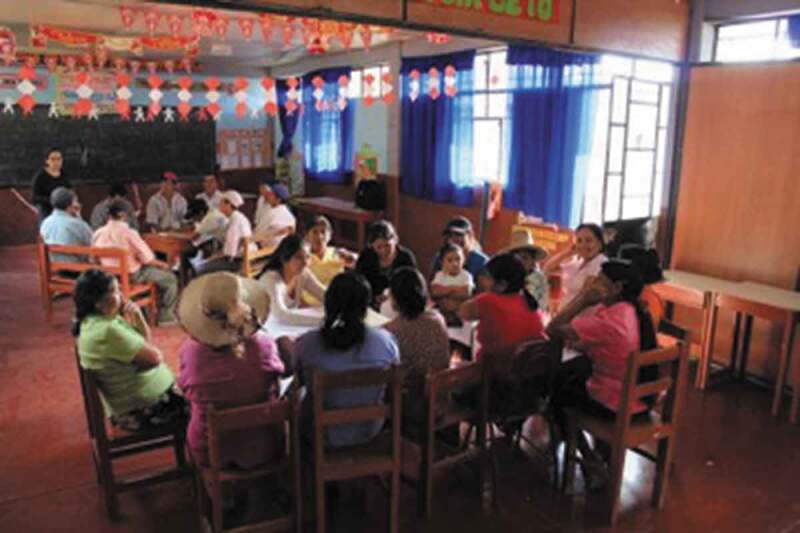
First consultation at micro level stakeholders

At the micro level, two groups of stakeholders participated, community and primary healthcare workers (PHCW). The first consultation at the micro level included presentations and discussions of preliminary findings and highlights from the formative studies, rather than a completed data analysis. The micro level meeting with community members also included discussions on definitions, causes, diagnosis, and management of chronic diseases. This was based on findings from the formative studies which highlighted limited knowledge related to the diseases and a request from the communities. After the explanation of each disease, participants broke out into smaller groups and discussed potential solutions to improve disease diagnosis and/or management (Figure 2). The process was repeated three times, one for each disease. The meeting with PHCW started with an explanation of the co-creation process followed by proposals for interventions or projects that could improve disease diagnosis and/or management in their community.

The first consultation resulted at the macro level with 84 ideas of projects or interventions from 23 participants. At the meso level, 10 ideas of projects or interventions from 6 participants were received. At the micro level, 30 ideas of projects or interventions were received from 68 participants (42 women and 22 men).

The major themes of the proposals included the following: health worker training; patient education to community members and children on the selected diseases; health resource management (human and material-medical devices, laboratory reagents-); water and sanitation improvement; access to healthcare, medicines, and diagnostic tests; policy interventions, financing and planning; telehealth; and activities with the community (e.g. bio-farming, health campaigns, improved pig rearing). Most of the proposals at the macro level were focused on policy, whereas most of the proposals at the meso level focused on training of health workers, policy (financing, planning) and health resources management. Proposals at the micro level were the training of health workers, health resources management and activities related to the community.

**Figure 3.**
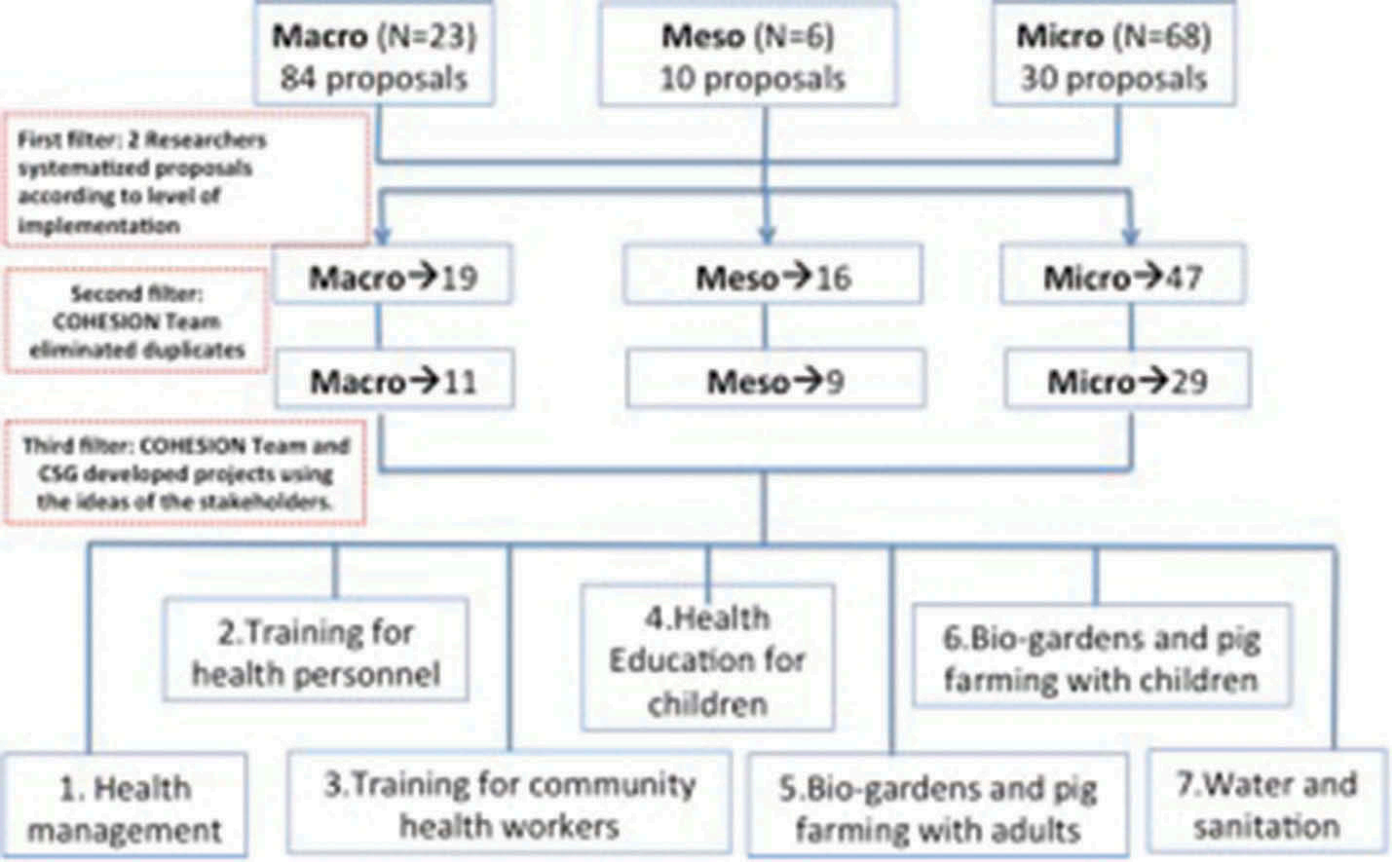
Process to filter the proposals of the first consultation

Consistent with the COHESION manual, the project team worked to prioritize and map out the list of interventions. This started with the elimination of duplicates followed by classifying them according to the level of implementation: policy, health system, or community level. Next, an additional activity took place which included a workshop with two researchers from Universidad Peruana Cayetano Heredia’s Global Health Center (Centro de Salud Global-CSG) in Tumbes, Peru, who had relevant experience in the region. During the workshop, feasibility and sustainability issues related to the proposals were discussed. A set of seven interventions were selected prior to the second consultation (see Figure 3).

N = number of participants, CSG: Centro de Salud Global.

### Second consultation

During the second consultation, each stakeholder group prioritized the list of interventions. At the macro and meso levels, stakeholders used the COHESION manual matrix where they scored proposed interventions according to the level of support, required and available resources, and possible impact [19]. At the micro level, participants prioritized the interventions according to what they felt was most important for their community.

The COHESION manual was adapted for the macro level participants based on learnings from the first consultation. Given the time constraints of this stakeholder group, a face to face meeting was replaced with email communication. The e-mail included information on the seven potential interventions, a table with evaluation criteria (support, resources and impact) and instructions on how to score each criterion. Twelve out of 23 participants answered the e-mail and thus follow-up emails were sent and then phone calls made to collect their answers.

For the meso level, the meeting started with an explanation of the co-creation process and the potential interventions. Participants completed the same table as the macro level and then discussed each intervention. Six people participated in the meeting and discussed the different options providing insights about the pros and cons of each.

At the micro level, 54 (40 women, 14 men) participated in the second consultation. First, potential interventions were described. Then, smaller groups were formed and each asked to select the three most important interventions, the three least important interventions, and one intervention in the middle. Notably, community members were very involved in the discussion and stressed that all the possible interventions would be important for their community.

The results of the second consultation are presented in Table 2. Training for community health workers and health personnel were listed as the most important interventions for the micro and macro levels, respectively. At the meso level, health management was rated highest.

**Table 2.** Prioritisation process after the second consultation

Micro^a^			
Community^a^	PHCW*	Meso^a^	Macro^a^
3.Training for community health workers	3.Training for community health workers	1. Health Management	2.Training for health personnel
2.Training for health personnel	5. Bio-gardens and pig farming in adults	4. Education for children	3.Training for community health workers
7.Water and sanitation	7.Water and sanitation	6. Bio-gardens and pig farming in children	7.Water and sanitation

^a^The list shows the interventions selected during the prioritisation process by each level.

PHCW: Primary healthcare workers.

After the two consultations; the COHESION Score [19] was applied by researchers to rank the seven interventions. The researchers have experience in fieldwork, from different disciplines (medicine, economics, anthropology and sociology), and academic degrees (PhD, MSc, Bachelor). The results are presented in Table 3 and show a maximum value of 100%, which corresponded to the training of community health workers. The minimum score obtained was 45%, which corresponded to child education. Using the information from the second consultation (Table 2) and the COHESION Score results (Table 3), three interventions were selected by the national research team. These included:

1. Training for community health workers: Training in health promotion and follow-up to patients with chronic conditions.
2. Capacity building for health personnel: Training using telemedicine and guidelines.
3. Health management: Training in health management to health personnel.

**Table 3.** Results of the COHESION score

COHESION Score						
100%	80%	80%	65%	60%	60%	45%
3.Training for community health workers	1. Health Management	7. Water and sanitation	6. Bio-gardens and pig farming in children	2.Training for health personnel	5. Bio-gardens and pig farming in adults	4. Education for children

In addition, improvement of water and sanitation systems were prioritised because they received a high COHESION Score. Ultimately, however, they were not included in the final project due to budget constraints and lack of researcher expertise in this area.

### Defining the interventions and a theoretical framework

Following the COHESION manual, the COHESION project team (Peru, Nepal, Mozambique and Switzerland) during a multi-country meeting used Hoffmann *et al*.’s 2014 TIDieR’s framework for ‘better reporting of interventions: template for intervention description and replication’ for each of the three interventions [22]. This specified the: what, why, who provided, how, and where [22].

At the end of the meeting, the potential interventions with the preliminary version of TIDieR were presented, and researchers received feedback from the advisory board.

A second multi-country meeting then took place to work on the intervention implementation protocol, better define the interventions (co-design), and decide if the interventions would be the same or different in the three countries.

The coordinators and research assistants from each country, one Principal Investigator, and two consultants with experience in the development of chronic disease interventions were present. During this meeting, researchers reviewed the preliminary results of the formative studies, discussed the study findings, re-visited the proposals and projects from the co-creation process. The study team realized the importance of having a theoretical framework to ground the interventions within. The project team adopted the World Health Organization’s Responsiveness Framework [23,24], which includes seven domains: dignity, confidentiality, autonomy, prompt attention, social support, basic amenities, and choice of provider. The concept of ‘responsiveness’, defined by the WHO ‘as the outcome that can be achieved when institutions and institutional relationships are designed in such a way that they are cognisant and respond appropriately to the universally legitimate expectations of individuals.’ This framework was found to be particularly relevant as it considers both the expectations of the people and health system actors about how the population should be treated, and the interactions that influence people’s experiences. The framework stresses that there is a positive association between health outcomes and responsiveness of a health system, and this includes non-medical aspects of health. The rationale for this selected framework was further rooted in the data from the project. Based on the formative research and existing evidence for the setting, it was evident that health systems and health services were weak. This framework also resonated with some of the guiding principles of the COHESION project, such as a: focus on the community and PHC; a change to the system; innovative approach; link with formative research and co-creation; focus on vulnerability, equity, social inclusion, gender; involvement of local government structures; decentralization; and scalability and sustainability.

### Co-design

The final phase in the COHESION manual is co-design. Co-design meetings were held to ensure that the views and needs of micro and meso level stakeholders were taken into consideration when designing the final intervention. Thus, all parties had a clear role in co-creating such solutions. This also helped to ensure the selected interventions met the criteria set out at the start regarding impact, population benefitting, feasibility, sustainability and scalability of interventions. During the co-design stage, the WHO Responsiveness Framework was presented to each group of stakeholders in separate meetings (community, health workers and regional stakeholders). The meso level included eight officials from the Regional Health Directorate. Two meetings with micro level stakeholders were held, one with 40 participants and the second with 46. Additionally, a meeting with one PHCW was held.

Participants from each level agreed with the selection of the WHO Responsiveness Framework and some from the meso level shared personal and work experiences related to the domains of the responsiveness framework. Participants in each meeting reviewed all responsiveness domains and selected the ones most important to them. Community members chose *prompt attention* and *dignity*, and PHCW selected *confidentiality* and *autonomy*. Community members chose *dignity* as a priority because they felt that PHCW sometimes questioned their actions. They chose *prompt attention* because sometimes the PHCW were not available in the health post, preventing their ability to receive medical care. At the meso level, all participants agreed that *clear communication* was a key element to address in communities. Participants mentioned that the low education level of the population and the poor communication skills of the health providers were the main factors in communication gaps. The other selected domains for most of the participants was *dignity*, followed by *autonomy*.

### Final selection of interventions and theory of change

The pathway from formative research to final interventions passing through co-creation is shown in Table 4.

**Table 4.** Responsiveness Framework project map

Responsiveness domain	Data from Formative Studies	Data from Co-creation process	Addressed in selected intervention activities
Clear Communication	- Communication about the diseases is not culturally adapted. (For example, informative brochures were delivered although there are illiterate people in the area). [HSA] - Diabetes is considered to be curable and hypertension is recognized as a chronic condition. [CHP] - For neurocysticercosis knowledge about transmission by consuming pork, lack of knowledge of the process and social stigma. [CHP]	Capacity Building for health workers Training of community health workers with focus on health promotion and follow-up of patients	Radio programs to motivate patients to clarify any concerns they may have about their health condition and treatment and to know more about diagnosis and management of chronic diseases. Capacity building on management of diabetes, hypertension and NCC, communication and dignity using role playing. Also, ‘Communication jar’
Prompt Attention	- Insufficient training and low self-perceived capability at PHC workers. [HSA] - The public health insurance does not cover all the expenses (e.g. transportation, lodging & meals when referral is needed). [HSA] - Long distance and high expenses to health facilities where diagnostic tests can be performed and treatment provided. [CHP]	Capacity Building to health workers Training of community health workers with focus on health promotion and follow-up of patients	Capacity building on management of diabetes, hypertension and NCC, communication and dignity using role playing. Also, ‘Communication jar’
Dignity	- Because of PHC workers lack of skills to treat patients with NCDs and NCC, they lack motivation to treat them. [HSA] - Patients do not find PHC workers at the health center during working hours. [HSA] - Lack of confidence in the skills of the health workers. [CHP]	-	Radio programs l to motivate patients to clarify any concerns they may have about their health condition and treatment and to know more about diagnosis and management of chronic diseases.
Autonomy	- Patients have little or no involvement in the care or treatment they are provided. [HSA]	Capacity Building to health workers	Capacity building on management of diabetes, hypertension and NCC, communication and dignity using role playing. Also, ‘Communication jar’
Confidentiality	- People avoided sharing with others that they or a relative had epilepsy (associated with witchcraft). [CHP]	Capacity Building to health workers	Capacity building on management of diabetes, hypertension and NCC, communication and dignity using role playing. Also, ‘Communication jar’
Quality of Basic Amenities			At the facility level a decentralised decision making on improving facility will be provided. This consist in a small grant to improve the PHC centre to buy material.

HSA: Health System Assessment, CHP: Community Health Perception, PHC: Primary Healthcare, NCD: Noncommunicable diseases, NCC: neurocysticercosis.

The interventions selected through the co-creation process in Peru include:

- At the community level: radio programs organized by young people that include interviews with community members, patients with chronic diseases, and health personnel. The aim is to motivate patients, to clarify any concerns they may have about their health condition and treatment, and to educate about the diagnosis and management of chronic diseases.
- At the level of health personnel: trainings to help in capacity building for management of diabetes, hypertension and neurocysticercosis. Communication and dignity to be addressed using role-playing as well as a communication tool called a ‘communication jar’ (a jar where with pre-selected questions about diagnosis or treatment). The aim is to empower patients to ask questions about health personnel and others that health personnel are trained to provide appropriate answers for.
- At the facility level: a decentralised decision-making framework to improve facilities. A small grant to improve the PHC infrastructure and/or equipment through tangible and measurable health facility improvements. These improvements fall under the responsiveness domain of the quality of basic amenities. The grant is for tangible health facility improvements (e.g. to paint the walls, to fix a roof, to buy medical equipment), not to hire new personnel or to pay existing health providers.

A final meeting of the project team was held to design the Theory of Change for the complex intervention. This work was based on the three initial interventions selected (Section 3.2). The training of health personnel was maintained, but training in soft skills was added; instead of the training of the community, radio programs were included; and finally, the health management component was replaced by a small grant for the health facility to be decided by both the community and health personnel on how best to use it.

**Figure 4.**
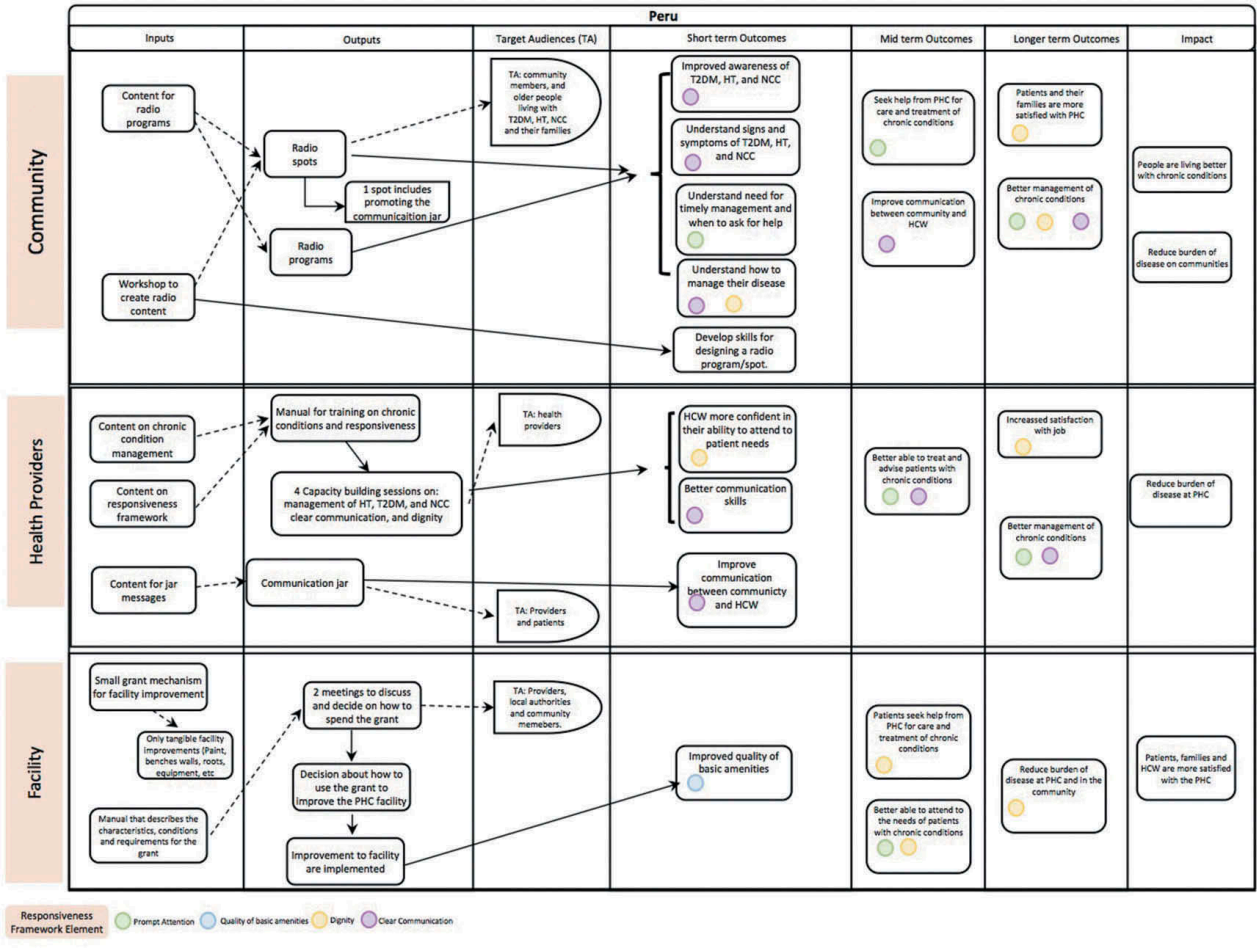
Theory of change for the intervention

The theory of change development forced the team to map all activities, inputs, target audiences, outputs, outcomes, and impacts of each component of the three levels of intervention. Each intervention activity was also linked to the WHO Responsiveness framework domains (see Figure 4). This tool will support the implementation of the project and in the process and summative evaluations.

## Discussion

This paper details that a process of co-creation is possible in a rural area of an upper-middle-income country involving a variety of stakeholders across different layers of the health system and moving beyond disease-specific programs and siloes. Through this process of co-creation, key lessons were identified. These include: the skillset of the research team; interactions with stakeholders; the importance of an advisory board; need for a theoretical framework; and the role of the funder.

For researchers, the process of co-creation was a challenge due to the investment of time and effort in comparison to other research approaches. Part of this was due to the multiple adaptations of the initial COHESION manual to make it feasible in the local context. One study found as challenge the time spent on the process, with 2 years invested to establish the project within the community [25]. This is similar to COHESION where 2.5 years were needed to complete the formative studies and the co-creation. Another challenge is the balance between scientific best practice versus the needs of the community [25].

Engaging stakeholders required both time and investment in developing the relationships as well as a set of skills in facilitation that are not necessarily a given to all researchers. The subjective ranking using the standardised tools included in the original manual of the interventions posed some difficulties for researchers with different backgrounds and perspectives. Overall having a multi-country team bringing together different skillsets and disciplines allowed mutual support throughout the project and sharing of lessons across the countries.

Another challenge was working with the stakeholders to generate ideas for interventions that could be feasible to implement. Some proposals from stakeholders were either too specific or outside of the scope of the project, e.g. related to communicable diseases, anaemia, or equipment for X-ray or ultrasounds. The investment of the stakeholder’s time is not negligible and must be considered when planning the multiple interactions needed in a co-creation and co-design process. Careful selection of participants is needed to ensure a diversity of views, especially in communities as well as being cognisant of different structures of power which could influence what stakeholders expressed, for example, giving equal weight to proposals from policy-makers versus the community.

One component of the COHESION Project that enabled local research teams to navigate the complexity of the issues was having a national advisory board for the project. This board provided feedback during different steps of the co-creation process, and gave support with their knowledge and expertise in the field. The challenge though with such a board was the availability of time of the individuals involved.

In the COHESION Project at the stage of developing interventions a framework was found and used to ground the overall work in a specific theory. This helped present the findings, the co-creation process and the final interventions in line with a given framework. Usually, researchers begin their overall project with a given framework which helps organize data from the formative studies as well as ‘sell’ the importance of their work to funders. With regards to funding, the COHESION project received support from the Swiss National Science Foundation and the Swiss Agency for Development and Cooperation under the Swiss Program for Research on Global Issues for Development to have a multi-staged project including formative work and intervention development.

These lessons are similar to five principles proposed by the UK’s National Institute for Health Research, which include: sharing power, including all perspectives and skills, respecting and valuing the knowledge of all those working together on the research, reciprocity, and building and maintaining relationships [26]. In order to accomplish these principles, it is important that researchers learn to change the conventional approach of identifying a problem, explore possible solutions from the literature review and select one (usually the more innovative) to prepare a proposal and get funding for its implementation [14]. However, overarching challenges remain with regards to how co-creation as part of the research process engages with policy-makers as well as how funding constrains researchers in implementing such an approach [27].

Our approach has some limitations, (i) we do not have a control group without co-creation or with another method to develop interventions, (ii) the approach was developed to be implemented in three countries and each country needed to adapt it, separately, (iii) the data from the formative studies were selected by the researchers and not by the stakeholders, so researchers could introduce bias in the selection of certain results (iv) the consultation meetings were conducted in different groups according to macro, meso and micro level, and this approach did not allow for interactions between different stakeholders, (v) researchers chose interventions based upon those that fit within the scope and timelines of the project; however, these are not necessarily what is best for the long term and sustainable development of the health system. There was an effort to find the right intersection of these needs (research vs sustainable services) in the interventions that were chosen. However, it is possible that better interventions were discarded due this. The use of the scoring system of the COHESION manual could be seen as restrictive, but, it was applied to ensure the feasibility and sustainability of the proposed interventions. The current Theory of Change did not involve the stakeholders, their input might be crucial in order to be more effective. Finally, the limitation of the process to a group of diseases challenge the ideal ‘true or pure’ co-creation where stakeholders can select the topic and use co-creation to improve their quality of life.

The strengths of this approach were that stakeholders were involved in the complete process of co-creation, not only in one piece of the project. This enabled communities to be key actors, policy-makers to have evidence appropriate to the context, health workers to be part of the decisions about which training is important and how it should be designed, and the community to take part in the decisions around the activities that will ultimately impact them. There is also the potential benefit that by involving the community this may impact the success of the intervention implementation.

## Conclusion

This study shows the implementation of a method to co-create interventions to address the complexity of chronic disease management at PHC in a rural area of an upper-middle-income country. It provides a practical example of the process of using co-creation to generate complex interventions and, in so doing, it fills a gap in the literature about this topic. Four main components, namely stakeholders, national research team, multi-country research team and advisory board, and framework selection were identified, as the lessons from this process. The lessons from this study will be useful to other researchers who want to follow this process of participative development of interventions.

## Data Availability

The study protocols are available online: www.cohesionproject.info

## Paper context

Current methodologies have failed to address the complexity of chronic conditions, co-creation is an option for the future development of sustainable and scalable interventions. For this reason, this study shows the implementation of a method to co-create interventions for chronic disease management at the primary health-care level.
